# Efficacy and safety of Jianzhong decoction in treating peptic ulcers: a meta-analysis of 58 randomised controlled trials with 5192 patients

**DOI:** 10.1186/s12906-017-1723-2

**Published:** 2017-04-14

**Authors:** Yan Sun, Jinping Zhang, Yuanyuan Chen

**Affiliations:** 1 Department of Chinese Medicine, Nankai Hospital, No. 6 Changjiang Road, Nankai District, Tianjin, 300100 China; 2 Department of Pharmacy, Affiliated Hospital of Logistics Institute of Chinese Armed Police Force, Tianjin, 300163 China

**Keywords:** Jianzhong decoction, Peptic ulcer, Randomised controlled trials, Meta-analysis

## Abstract

**Background:**

Jianzhong decoction is widely used to treat peptic ulcers; however, due to lack of systematic evaluations, its clinical efficacy remains controversial. We performed meta-analysis to evaluate the efficacy and safety of Jianzhong decoction in treating peptic ulcers.

**Methods:**

Studies were systematically retrieved from PubMed, Embase, Cochrane library, China National Knowledge Infrastructure, Wanfang Database, Chongqing VIP, China Biology Medicine disc (CBMdisc), and references cited in related studies/reviews. Extracted data included the total effective rate, *helicobacter pylori* eradication rates, recurrence rate, and adverse reaction rate. Fifty-eight randomised controlled trials involving 5192 patients were included in the final analysis.

**Results:**

Results showed that Jianzhong decoction therapy was more effective than conventional Western medicine therapy (total effective rate, odds ratio [OR] = 4.29, 95% confidence interval [CI]: 3.51–5.23, *P* = 0.000; *helicobacter pylori* eradication rates, OR =2.10, 95% CI: 1.69–2.61, *P* = 0.000; recurrence rate, OR =0.23, 95% CI: 0.18–0.29, *P* = 0.000; and adverse reaction rate, OR =0.20, 95% CI: 0.12–0.33, *P* = 0.000).

**Conclusions:**

Jianzhong decoction increased the total effective rate and *helicobacter pylori* eradication rate, and lowered the recurrence and adverse reaction rates. The results of this study can be used as a guide for clinical treatment of peptic ulcers.

**Electronic supplementary material:**

The online version of this article (doi:10.1186/s12906-017-1723-2) contains supplementary material, which is available to authorized users.

## Background

Peptic ulcer (PU) refers to the ulcer in the gastrointestinal (GI) mucosa occurring due to the erosion induced by gastric acid and pepsin. Gastric and duodenal ulcers are the most commonly seen PUs in clinical practice. Typically, PU has the unique characteristics of self-healing and recurrence, as well as a relatively high incidence rate with up to a 50% short-term incidence rate [1]. Moreover, PU has a complicated pathogenesis, including excessive gastric acid secretion, the necessitation of non-steroidal anti-inflammatory drugs (NSAIDs), *Helicobacter pylori* (Hp) infection, and gastric mucosal injury [2]. Currently, PU is mainly treated with the triple or quadruple therapy of Western medicine (WM), which eradicates Hp and protects the gastric mucosa by inhibiting gastric acid secretion. Patients treated with WM show significant symptomatic improvements, but have a relatively higher short-term recurrence rate and a poor long-term therapeutic efficacy. Additionally, patients become susceptible to adverse reactions during WM treatment [2, 3]. Traditional Chinese medicine (TCM) and integrated medicine are the current focus of research addressing the PU treatment. In the TCM theory, middle-jiao (the middle part of a human being) is a key location for human digestion. And many digestive painful diseases can be treated by Jianzhong decoction. The name, Jianzhong, means construct the qi of middle-jiao. Clinically, TCM uses some combinations of sweet and warm-natured herbs as Jianzhong decoction. Here, the main herbs in the decoction are Ginger, Cassia twig, Radix paeoniae alba, Jujube and Licorice. Previous studies have confirmed that Jianzhong decoction has been widely applied in the clinical treatment of PU using various prescriptions; however, their therapeutic efficacies are still controversial [4]. Several studies have shown that Jianzhong decoction outperforms the conventional WM in terms of therapeutic efficacy against PU with a high healing rate, low recurrence rate, high Hp eradication rate, and a significantly reduced ulcer area. However, other studies showed that Jianzhong decoction has no significant advantage over the conventional WM in treating PU with respect to the total effective rate [5, 6]. Therefore, our study is aimed at gathering randomised controlled trials (RCTs) that involved Jianzhong decoction in PU treatment, in order to systematically evaluate its therapeutic efficacy and safety and provide the basis for clinical PU treatment.

## Methods

### Literature search

PubMed, Embase, Cochrane library, China National Knowledge Infrastructure (CNKI), Wanfang Database, China Science and Technology (CSTJ) Database (Chongqing VIP), and China Biology Medicine disc (CBMdisc) were subjected to computational searches up until November 16, 2016. The Chinese search keywords were Jianzhong decoction, PU, gastric ulcer, duodenal ulcer, ulcerative colitis, and Crohn’s disease. The English search keywords were Jianzhong decoction, Jianzhong tang, Jianzhong, peptic ulcer, gastric ulcer, duodenal ulcers, digestibility ulcer, ulcer, peptic colitis, Crohn’s disease, and clinical. In addition, we further searched the references cited in relevant literature reviews in order to avoid any oversights.

### Literature inclusion and exclusion criteria

Inclusion criteria were: (1) Research Type: Randomized Controlled Trials (RCTs); (2) patients in the treatment group were treated with mainly Jianzhong decoction or Jianzhong decoction integrated with WM and/or other treatment(s); while patients in the control group were treated with placebo or conventional WM; and (3) the outcome indicators included at least one of the following: total effective rate, Hp eradication rate, recurrence rate, and/or adverse reactions.

Exclusion criteria were: (1) irrelevant studies; (2) animal studies; (3) literature reviews; (4) case and expert reports; (5) non-RCTs; (6) studies that did not use Jianzhong decoction in treating PU; (7) studies related to GI bleeding, pyloric obstruction, and other severe complications; (8) studies that used Jianzhong decoction as adjuvant therapy; (9) duplicate publications; (10) Jianzhong prescription in non- decoction; and (11) No desired outcomes (studies providing no extractable data).

### Literature selection and data extraction

The titles and abstracts of the retrieved articles were read independently by two researchers. After excluding the articles that did not meet the inclusion criteria, full texts of the remaining articles were perused in order to validate their inclusion. Any disagreement regarding the inclusion of articles was solved via discussion or decided by a third researcher. Both researchers independently extracted the data from the included studies. These included the names of the authors, year of publication, study samples, interventional measures of the experimental and control groups, efficacy evaluation indicators, treatment course, type of ulcer, follow-up duration, randomisation methods, baseline equilibrium, blinded experiment, and results.

### Research quality evaluation

The methodological quality of the included studies was evaluated using the modified Jadad scale [7]. The evaluated items included the random sequence generation, allocation concealment, blinded experiment, and lost to follow-up. The range of total RCT scores were 1–7, wherein, studies with scores of 1–3 were considered of low quality while studies with scores of 4–7 were considered of high quality. The evaluation of data quality was performed independently by two evaluators and any disagreement was resolved via discussion.

### Statistical analysis

The meta-analysis was performed using the Stata statistical software (Version 12.0). The count data were expressed as odds ratio (OR) and 95% confidence intervals (CIs). The *I* ^*2*^ index was used to describe the heterogeneity among studies; wherein *I* ^*2*^ < 50% denotes the absence of heterogeneity among studies that were analysed using the fixed effects model whereas studies with *I* ^*2*^ > 50% were analysed using the random effects model. Potential publication bias was analysed using the funnel plot, Egger’s test, and Begg’s test. The stability of the results was evaluated using the sensitivity analysis, where all included studies were excluded one by one and the remaining studies were subjected to meta-analysis again to check for any change in the results before and after the exclusion. Based on the assumption that publication bias leads to asymmetry in funnel plots, we further analysed the robustness of the results using the non-parametric trim and filling method. Statistical significance was considered at *P* < 0.05.

## Results

### Search results and the general situation of included studies

**Fig. 1:**
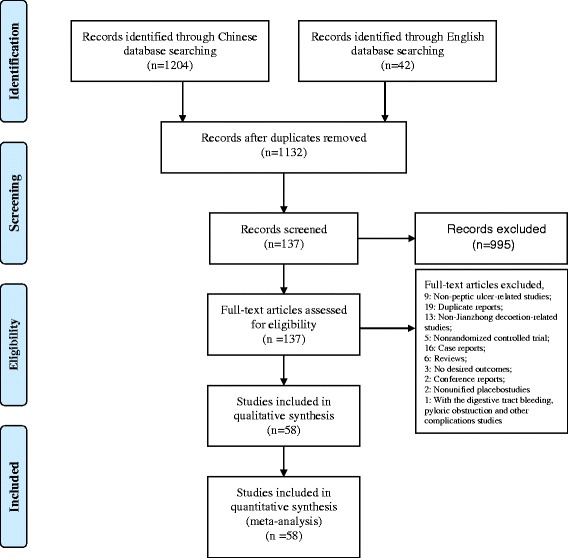
PRISMA 2009 Flow Diagram

The workflow of the literature search and selection is illustrated in Fig. 1. A total of 1246 articles consisting of 42 English articles and 1204 Chinese articles were retrieved according to the search strategy and data collection method. A total of 114 duplicate publications were excluded. Moreover, a total of 137 articles were preliminarily included after reading the titles and abstracts. We further examined the full texts of these 137 articles and excluded nine non-PU articles, 19 duplicate publications, 13 non-Jianzhong decoction articles, five non-RCTs, 16 case reports, six literature reviews, three articles with no extractable data, two conference papers, two articles with inconsistent control drugs, and one article reporting GI bleeding, pyloric obstruction, and other complications. We finally included a total of 58 articles, all of which were published in China [5, 6, 8–63]. The basic characteristics of these included studies are shown in Table 1. Briefly, these 58 studies were divided into a number of categories. Twenty-one studies used Huangqi Jianzhong decoction or Huangqi Jianzhong decoction combined with other prescriptions or therapies and WM [13–15, 18, 20, 23, 31, 34, 37, 38, 40, 42, 43, 48, 49, 51–54, 58, 60, 63]. Sixteen studies used Xiaojianzhong decoction alone or Xiaojianzhong decoction combined with other prescriptions and WM [5, 6, 8, 9, 12, 17, 19, 21, 25, 27, 28, 30, 35, 36, 39, 41]. Eleven studies used Huangqi Jianzhong decoction alone or Huangqi Jianzhong decoction combined with other prescriptions [24, 32, 45, 46, 50, 55, 57, 59, 61, 62]. Nine studies used Xiaojianzhong decoction alone or Xiaojianzhong decoction combined with other prescriptions [10, 11, 16, 22, 26, 29, 33, 44, 55]. A total of 33 studies clearly stated the type of PU studied, which mainly included gastric ulcer, duodenal ulcer, or the combination of both [9, 15, 18, 20, 24, 27, 31–39, 41, 42, 45, 47–50, 52, 53, 55–63], while other studies did not clearly state the type of the ulcer. The included studies recruited a total of 5192 patients, consisting of 2801 patients from the treatment group and 2391 patients from the control group.

**Table 1:** Characteristics of the included studies.

Study ID	Sample size (*n*/*n*) Treatment vs Control	Sex(M/F)	Age, y	Treatment intervetion	Control intervetion	Outcomes	Type of PU GU/DU/CU	Time of Treatment	Follow-up duration	Jadad score	Methodology of RCT	Analysis of baseline balance	Blinding
Nie JT 2016 [8]	75/75	25/50:29/46	44. 7 ± 5. 2:45. 1 ± 4. 9	Xiaojianzhong decoction + liangfu wan + omeprazole + amoxicillin	Omeprazole +amoxicillin	Total effective rate	−	4w	2w	1	Words of random	Comparability	No
Shi ZG 2016 [5]	66/66	34/32:35/31	38 ± 8.0:37.6 ± 7.9	Xiaojianzhong decoction + omeprazole +amoxicillin + clarithromycin + clavulanate potassium	Omeprazole +amoxicillin + clarithromycin + clavulanate potassium	Hp eradication rates; recurrence rate	−	4w	6w	2	Random number table	Comparability	No
Guo RP 2016 [9]	78/72	42/36:40/32	44.3:42.6	Xiaojianzhong decoction + Jiawei Danshen decoction + omeprazole + clarithromycin + amoxicillin	Omeprazole + clarithromycin + amoxicillin	Total effective rate; Hp eradication rates; recurrence rate; adverse reaction rate	19/49/10:18/46/8	6w	6mo	1	Words of random	Comparability	No
Xie DM 2016 [10]	34/34	16/19:20/14	38.92 ± 4.26:39.15 ± 4.19	Jia wei Xiaojianzhong decoction	Omeprazole	Total effective rate; adverse reaction rate	−	8w	−	2	Random number table	Comparability	No
Cai QP 2016 [11]	36/36	22/14:20/16	42.8 ± 4.6:44.0 ± 5.5	Xiaojianzhong decoction	Omeprazole + amoxicillin	Total effective rate	−	4w	−	1	Words of random	Comparability	No
Li JL 2016 [12]	34/34	21/13:20/14	42.58 ± 4.49:41.96 ± 4.88	Xiaojianzhong decoction + esomeprazole + amoxicillin + clarithromycin	Esomeprazole + amoxicillin + clarithromycin	Total effective rate; Hp eradication rates; recurrence rate	−	4w	1y	2	Random number table	Comparability	No
Li J 2015 [13]	43/43	22/21:25/18	60.13 ± 2.79/58.12 ± 1.95	Huang qi jiangzhong decoction jia jiang + omeprazole + clarithromycin + amoxicillin	Omeprazole + clarithromycin + amoxicillin	Total effective rate; recurrence rate; adverse reaction rate	−	8w	6mo	1	Words of random	Comparability	No
Yang CY 2015 [14]	87/87	46/41:49/38	44.9 ± 3.9:47.4 ± 3.5	Huang qi jiangzhong decoction jia jiang + ranitidine	Ranitidine	Total effective rate; recurrence rate; Hp eradication rates; adverse reaction rate	−	4w	−	1	Words of random	Comparability	No
Wang HX 2015 [15]	33/33	41/25	40.4 ± 8.6	Huang qi jiangzhong decoction + omeprazole + amoxicillin + clarithromycin + hydrotalcite	Omeprazole + amoxicillin + clarithromycin + hydrotalcite	Total effective rate; recurrence rate	26/32/10	6w	1y	2	Method of random data	Comparability	No
Li X 2015 [16]	50/50	24/26:23/27	50.0 ± 0.5:50.0 ± 0.3	Xiaojianzhong decoction	Omeprazole	Total effective rate; adverse reaction rate	−	8w	−	1	Words of random	Comparability	No
Yang ZS 2015 [17]	75/75	25/50:29/46	44.7 ± 5. 2:45.1 ± 4.9	Xiaojianzhong decoction + liangfu wan + omeprazole + amoxicillin	Omeprazole + amoxicillin	Total effective rate	−	4w	−	1	Words of random	Comparability	No
Han B 2015 [18]	28/28	18/10:19/9	33.8 ± 11.8:34.2 ± 12.5	Jia wei Huang qi jiangzhong decoction + omeprazole + clarithromycin + amoxicillin	Omeprazole + clarithromycin + amoxicillin	Hp eradication rates	0/28/0:0/28/0	3w	−	2	Random number table	Comparability	No
Wen LQ 2015 [19]	40/40	27/13:26/14	44.37 ± 4.18:45.62 ± 4.25	Xiaojianzhong decoction + omeprazole + amoxicillin	Omeprazole + amoxicillin	Hp eradication rates; recurrence rate	−	4w	1y	2	Random number table	Comparability	No
Gao SJ 2015 [20]	50/50	24/26:23/27	45.7 ± 5.4:45.2 ± 5.0	Huang qi jiangzhong decoction jia jiang + omeprazole + hydrotalcite + amoxicillin	Omeprazole + hydrotalcite + amoxicillin	Total effective rate; recurrence rate; adverse reaction rate	19/31/0:18/32/0	6w	6mo	1	Words of random	Comparability	No
Zhang JJ 2014 [21]	31/31	23/8:20/11	40.2 ± 2.9:1.8 ± 1.7	Xiaojianzhong decoction + liangfu wan + omeprazole + clarithromycin + amoxicillin	Omeprazole + clarithromycin + amoxicillin	Total effective rate	−	2w	4mo	1	Words of random	Comparability	No
Xu Y 2014 [22]	36/36	22/14:13/23	47.3:37.2	Jia wei Xiaojianzhong decoction	Omeprazole	Total effective rate	−	40d	−	0	Registration order	Comparability	No
Liu CY 2014 [23]	56/56	39/17:37/19	71. 68 ± 8. 37:69. 52 ± 7. 63	Huang qi jiangzhong decoction(Mianjian granule) + rabeprazole	Rabeprazole	Total effective rate	−	4w	−	2	Random number table	Comparability	No
Wu QM 2014 [24]	25/25	28/22	40.3	Huang qi jiangzhong decoction	Omeprazole + metronidazole +amoxicillin	Total effective rate; recurrence rate	25/0/0:25/0/0	8w	1y	1	Words of random	Comparability	No
Gan GC 2014 [25]	53/53	29/24:28/25	39.4 ± 5.6:40.26 ± 5.1	Jia wei Xiaojianzhong decoction + Omeprazole + clarithromycin + amoxicillin	Omeprazole + clarithromycin + amoxicillin	significant efficiency rate; recurrence rate	−	4w	1y	1	Words of random	Comparability	No
Xi SY 2013 [26]	62/20	38/24:12/8	19 ~ 62:21 ~ 61	Jia wei Xiaojianzhong decoction	Omeprazole	Total effective rate; adverse reaction rate	−	8w	−	2	Random number table	Comparability	No
Fan WH 2013 [27]	56/56	32/24:30/26	47.18 ± 11. 70:46. 29 ± 11. 30	Xiaojianzhong decoction + esomeprazole + amoxicillin/ clarithromycin + barking dogs wow ketone	Esomeprazole + amoxicillin/ clarithromycin + barking dogs wow ketone	Total effective rate; Hp eradication rates; recurrence rate	14/32/10:22/28/16	8w	1y	2	Random sequence and registration order	Comparability	No
Yu B 2013 [28]	49/49	67/31	36.24 ± 4.12	Xiaojianzhong decoction + liangfu wan + omeprazole + clarithromycin + amoxicillin + ranitidine	Omeprazole + clarithromycin + amoxicillin + ranitidine	Total effective rate; Hp eradication rates; recurrence rate	−	8w	6mo	2	Draw lots	Comparability	No
Li SH 2013 [29]	68/68	39/29:36/32	43.4 ± 5.5:42.6 ± 4.5	Jia wei Xiaojianzhong decoction	Omeprazole + amoxicillin	Total effective rate; recurrence rate	−	4w	1y	1	Words of random	Comparability	No
Wan YH 2013 [30]	37/37	19/18:20/17	36.58 ± 3.36:37.81 ± 3.64	Xiaojianzhong decoction jia jian + omeprazole + clarithromycin + amoxicillin	Omeprazole + clarithromycin + amoxicillin	Total effective rate; Hp eradication rates; recurrence rate; adverse reaction rate	−	4w	6mo	2	Random number table	Comparability	No
Wu YJ 2013 [31]	34/34	42/26	40.1 ± 5.8	Huang qi jiangzhong decoction + omeprazole + barking dogs wow ketone + clarithromycin	Omeprazole + barking dogs wow ketone + clarithromycin	Total effective rate; Hp eradication rates; adverse reaction rate	0/34/0:0/34/0	4w	−	1	Words of random	Comparability	No
Si AJ 2013 [32]	29/29	32/26	40.3	Huang qi jiangzhong decoction	• Omeprazole + metronidazole +amoxicillin	Total effective rate; recurrence rate	29/0/0:29/0/0	8w	1y	1	Words of random	Comparability	No
Wu WG 2013 [33]	35/22	28/29	24 ~ 57	Xiaojianzhong decoction jia jian	Omeprazole + metronidazole + amoxicillin + clarithromycin	Total effective rate	22/21/14	4w	−	1	Words of random	Comparability	No
Bi MY 2013 [34]	32/31	20/12:19/12	34.7:36.2	Huang qi jiangzhong decoction +rabeprazole sodium + hydrotalcite	• Rabeprazole sodium + hydrotalcite	Total effective rate; Hp eradication rates	10/19/3:8/21/2	6w	−	1	Words of random	Comparability	No
Li RD 2012 [35]	30/30	16/14:17/13	34.48 ± 2.46:34.67 ± 2.28	Xiaojianzhong decoction + omeprazole + amoxicillin	Omeprazole + amoxicillin	Total effective rate; Hp eradication rates; recurrence rate; adverse reaction rate	8/18/4:9/16/5	4w	1y	1	Words of random	Comparability	No
Zou YQ 2012 [36]	45/45	28/17:31/14	45.2 ± 5.1:43.2 ± 5.0	Jia wei Xiaojianzhong decoction + lansoprazole +amoxicillin/clarithromycin + guojiaobi	Lansoprazole +amoxicillin/clarithromycin + guojiaobi	Total effective rate	10/22/13:14/20/11	24w	−	2	Random number table	Comparability	No
Deng YX 2012 [37]	38/30	−	−	Huang qi jiangzhong decoction + ranitidine	Ranitidine	Total effective rate; Hp eradication rates	5/33/0:3/27/0	4w	−	1	Words of random	Comparability	No
Li XJ 2012 [38]	46/40	29/17:25/15	35.9 ± 11.8:34.1 ± 1.21	Huang qi jiangzhong decoction + omeprazole + clarithromycin + amoxicillin	• Omeprazole + clarithromycin + amoxicillin	Hp eradication rates	0/46/0:0/40/0	1w	6w	1	Words of random	Comparability	No
Cao AY 2011 [39]	72/72	45/27:47/25	37:36	• Xiaojianzhong decoction jia jiang + omeprazole + metronidazole +amoxicillin + clarithromycin	Omeprazole + metronidazole + amoxicillin + clarithromycin	Total effective rate	25/47/0:28/44/0	8w	−	1	Words of random	Comparability	No
Dan JK 2011 [40]	30/30	21/9:23/7	小于30,30 ~ 50,大于50	Huang qi jiangzhong decoction + shixiaosan + omeprazole/omeprazole + amoxicillin + clarithromycin	Omeprazole/Omeprazole + amoxicillin + clarithromycin	Total effective rate; Hp eradication rates	−	6w	−	1	Words of random	Comparability	No
Hu J 2011 [41]	75/25	49/26:18/7	18 ~ 65:20 ~ 61	Jia wei Xiaojianzhong decoction + esomeprazole + amoxicillin/ clarithromycin + furazolidone	Ranitidine + bismuth potassium citrate	Total effective rate; adverse reaction rate	10/38:10:9/13/3	8w	−	0	Registration order	Comparability	No
Jang YH 2011 [42]	65/65	36/29:37/28	38.2 ± 7.8:37.9 ± 8.1	Huang qi jiangzhong decoction + ranitidine	Ranitidine	Total effective rate; recurrence rate	20/33/12:22/32/11	4w	1y	1	Words of random	Comparability	No
Yi LH 2011 [43]	50/50	32/18:23/27	45.9:48.5	Huang qi jiangzhong decoction + omeprazole + amoxicillin + clarithromycin	Omeprazole + amoxicillin + clarithromycin	Total effective rate	−	4w	−	2	Random number table	Comparability	No
Wu GL 2010 [44]	45/45	26/19:28/17	59.34 ± 21.65:56.99 ± 16.06	Jia wei Xiaojianzhong decoction	Omeprazole/Omeprazole + amoxicillin	Effective rate; recurrence rate	−	4w	1y	2	Random number table	Comparability	No
Wu LN 2010 [45]	40/32	23/17:18/14	44.6:43.50	Jia wei Huang qi jiangzhong decoction	Omeprazole + furazolidone + clarithromycin	Total effective rate; Hp eradication rates; recurrence rate; adverse reaction rate	18/22:14/18	4 or 6w	1y	1	Words of random	Comparability	No
Li XS 2010 [46]	45/42	−	51 ± 3.4:49 ± 4.8	Huang qi jiangzhong decoction jia jiang + acupoint application	Omeprazole + metronidazole + amoxicillin	significant efficiency rate; recurrence rate; Hp eradication rates; adverse reaction rate	−	4w	1mo	0	Registration order	Comparability	No
Zhu XX 2009 [6]	22/24	15/7:18/6	30 ~ 58:28 ~ 56	Jia wei Jiangzhong decoction + omeprazole + amoxicillin + clarithromycin	Omeprazole + amoxicillin + clarithromycin	Total effective rate	−	8w	−	1	Words of random	Comparability	No
Cui BS 2009 [47]	35/35	21/14:23/12	17 ~ 75:18 ~ 74	Jia wei Huang qi jiangzhong decoction + clarithromycin + amoxicillin + omeprazole	Omeprazole + amoxicillin + clarithromycin	Total effective rate; adverse reaction rate	20/15/0:22/13/0	4w	−	0	Registration order	Comparability	No
Gao Y2009 [48]	62/60	48/14:47/13	43.5:44.6	Huang qi jiangzhong decoction + famotidine + tinidazole/famotidine + tinidazole + amoxicillin	Famotidine + tinidazole/famotidine + tinidazole + amoxicillin	Total effective rate	9/48/5:7/47/6	6w	−	1	Words of random	Comparability	No
Zhan SL 2009 [49]	60/36	42/18:24/12	8 ~ 65:11 ~ 55	Jia wei Huang qi jiangzhong decoction	Ranitidine	Total effective rate; Hp eradication rates; adverse reaction rate	21/31/8:13/19/4	8w	−	0	Registration order	Comparability	No
Li YL 2009 [50]	40/34	27/13:22/12	43:42	Huang qi jiangzhong decoction + omeprazole + amoxicillin + metronidazole	Omeprazole + amoxicillin + metronidazole	Total effective rate; Hp eradication rates	18/22:16/18	4w	−	1	Words of random	Comparability	No
Pan DJ 2008 [51]	39/39	47/31	36.5	Huang qi jiangzhong decoction + omeprazole + amoxicillin + clarithromycin	Omeprazole + amoxicillin + clarithromycin	Total effective rate; adverse reaction rate	−	4w	−	1	Words of random	Comparability	No
Nong ZX 2008 [52]	40/40	25/15:23/17	25.21 ± 2.32:23.21 ± 2.98	Huang qi jiangzhong decoction jia jiang + omeprazole + amoxicillin + tinidazole	Omeprazole + amoxicillin + tinidazole	Total effective rate; recurrence rate; Hp eradication rates	13/20/5:10/25/4	8w	1y	0	Registration order	Comparability	No
Zhou XL 2008 [53]	45/45	24/21:27/18	19 ~ 62:18 ~ 64	Jia wei Huang qi jiangzhong decoction + omeprazole + ranitidine + aluminum hydroxide piece	Omeprazole + ranitidine + aluminum hydroxide piece	Total effective rate	5/39/1:6/37/2	4w	−	1	Words of random	Comparability	No
Zhao XL 2008 [54]	120/60	76/44:44/16	41.9 ± 8.9:43.0 ± 8.3	Huang qi jiangzhong decoction jia jiang	Ranitidine	Total effective rate; Hp eradication rates;adverse reaction rate	−	4w	−	0	Registration order	Comparability	No
Huang H 2007 [55]	25/25	15/10:18/7	43.5 ± 6.5:38.4 ± 6.7	Jia wei xiaojianzhong decoction	Omeprazole + amoxicillin + metronidazole	Total effective rate; recurrence rate; Hp eradication rates	6/12/7:9/13/3	4w	1y	2	Random number table	Comparability	No
Wang ZC 2007 [56]	40/40	28/12:27/13	41 ± 1.2:40 ± 1.4	Jia wei Huang qi jiangzhong decoction	• Famotidine + amoxicillin + tinidazole + guojiaobi	Total effective rate; Hp eradication rates; adverse reaction rate	16/24/0:16/24/0	4w	−	0	Registration order	Comparability	No
Gao XX 2006 [57]	80/80	66/14:60/20	2 ~ 72:22 ~ 68	Jia wei Huang qi jiangzhong decoction + omeprazole + amoxicillin + bismuth potassium pitrate	Omeprazole + amoxicillin + bismuth potassium pitrate	Total effective rate	0/80/0:0/80/0	4w	−	1	Words of random	No	No
Chen YJ 2005 [58]	30/30	17/13:20/10	45.63 ± 15.1:44.82 ± 18.0	Huang qi jiangzhong decoction	Ranitidine	Total effective rate	30/0/0:30/0/0	4w	−	1	Words of random	Comparability	No
Tang QJ 2002 [59]	20/20	12/8:13/7	36.0 ± 2.4:37.0 ± 1.8	Jia wei Huang qi jiangzhong decoction	Gentamicin + cimetidine	Total effective rate	0/20/0:0/20/0	1w	−	1	Words of random	Comparability	No
Chai LM 2001 [60]	56/28	31/25:16/12	35.1 ± 8.2:33.9 ± 9.8	Huang qi jiangzhong decoction + ranitidine	Ranitidine	Total effective rate; recurrence rate	15/29/12:8/14/6	4w	1y	1	Words of random	Comparability	No
Hou AQ 2001 [61]	100/100	58/42:52/48	45:50	Jia wei Huang qi jiangzhong decoction	Amoxicillin + guojiaobi + cimetidine	Total effective rate	44/36/16:51/42/7	2w	1y	1	Words of random	Comparability	No
Qian CY 2000 [62]	24/24	16/8:17/7	40:42	Huang qi jiangzhong decoction	Ranitidine + amoxycillin + metronidazole	Hp eradication rates; recurrence rate; adverse reaction rate	0/24/0:0/24/0	2w	1y	1	Words of random	Comparability	No
Chen HX 1997 [63]	50/50	30/20:30/20	−	Huang qi jiangzhong decoction + omeprazole	Omeprazole	recurrence rate	0/50/0:0/50/0	4w	1y	1	Words of random	No	No

*Hp* helicobacter pylori, *w* weeks, *mo* month, *y* year, *M* male, *F* female, *PU* peptic ulcer, *GU* gastric ulcer, *DU* duodenal ulcer, *CU* complex ulcer, *RCT* randomized controlled trial

### Evaluation of the methodological quality of included studies

Among those 58 studies, two studies did not specify the baseline equilibrium [57, 63], while the remaining 56 studies mentioned that the treatment and control groups were “comparable”. There were 24 studies that did not clearly specify the diagnostic criteria [11–13, 15, 17, 18, 24, 28, 31–33, 36–39, 51–54, 57, 62, 63], while the remaining 34 studies have clearly specified the diagnostic criteria. There were three studies [6, 36, 62] that only defined the inclusion criteria and five that only defined the exclusion criteria [12, 22, 29, 34, 35]; while the remaining 40 studies did not clearly define the inclusion and exclusion criteria. There were 11 studies that adopted the random number table method [5, 10, 12, 18, 19, 23, 26, 30, 36, 43, 44, 55]. Three studies used the random data method [15], the random sequence method [27], or the lottery method [28], whereas eight studies randomised according to the admission order [23, 41, 46–48, 52, 54, 56]. The remaining 36 studies only mentioned the term “random” in their articles. None of the studies mentioned using blinded methods. Only one study mentioned the withdrawal of a patient [16], while the remaining studies did not mention patients who became lost to follow-ups. There were in total 28 follow-up studies; eighteen 1-year follow-up studies [12, 15, 19, 24, 25, 27, 29, 32, 35, 42, 44, 45, 52, 55, 60–63], five 6-month follow-up [9, 13, 20, 28, 30], one 4-month follow-up [24], one 1-month follow-up [46], two 6-week follow-up [5, 38], and one 2-week follow-up study [8]. All of the studies had carried out a thorough analysis on the recurrence rate. In one study that has analysed the recurrence rate [57], we failed to retrieve the recurrence rate of treatment and control groups. Another study has analysed the recurrence rate, but did not clearly stated the follow-up duration [8]. According to the Jadad scale, there were 15 studies that scored 2-points [5, 10, 12, 15, 18, 19, 23, 26–28, 30, 36, 43, 44, 55] and 35 studies that scored 1-point, while the remaining eight studies scored 0-point, indicating that these RCTs have low methodological quality (See Table 1.).

### Results of meta-analysis

**Fig. 2:**
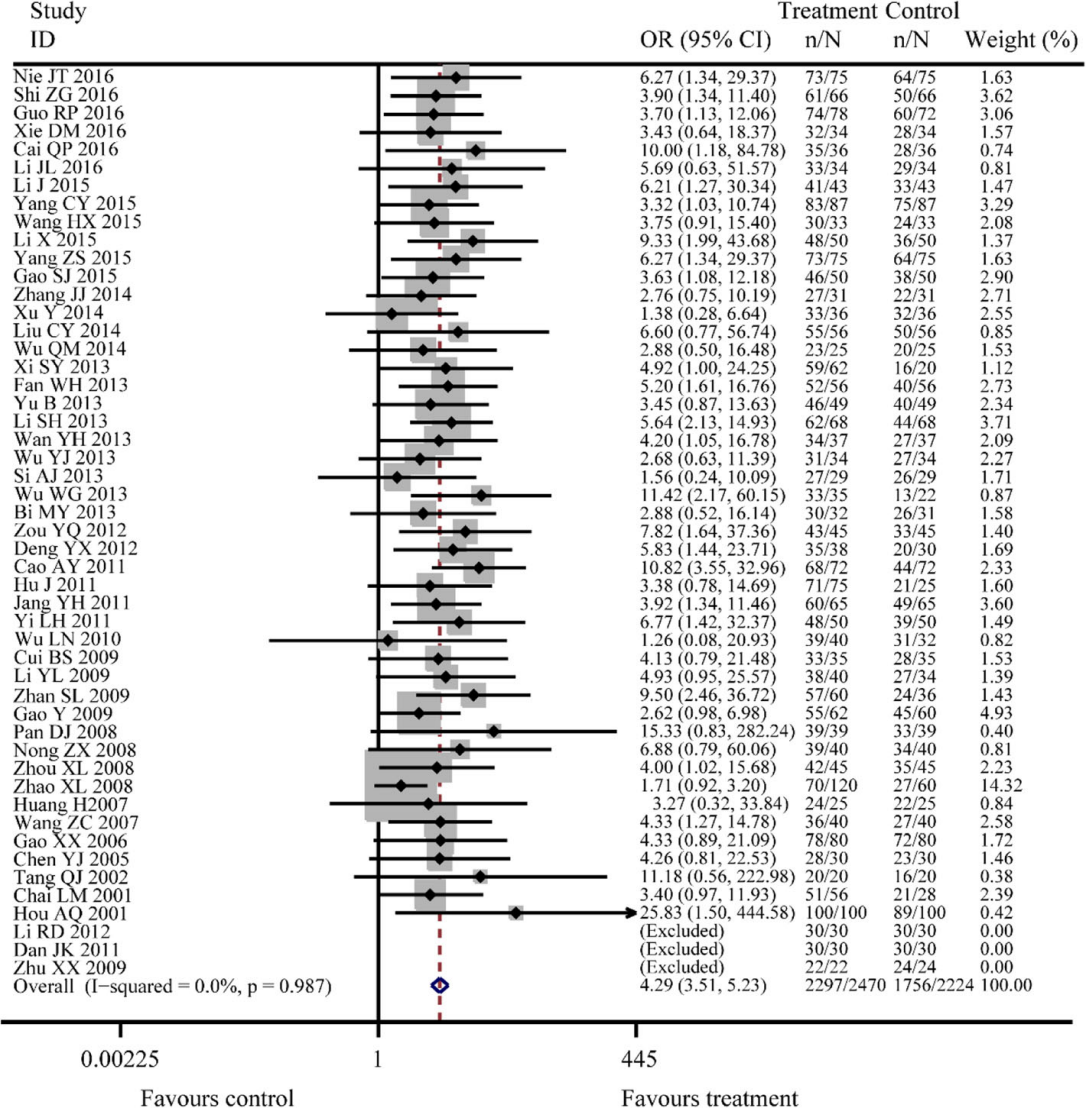
Meta-analysis of total effective rates about two groups

**Fig. 3:**
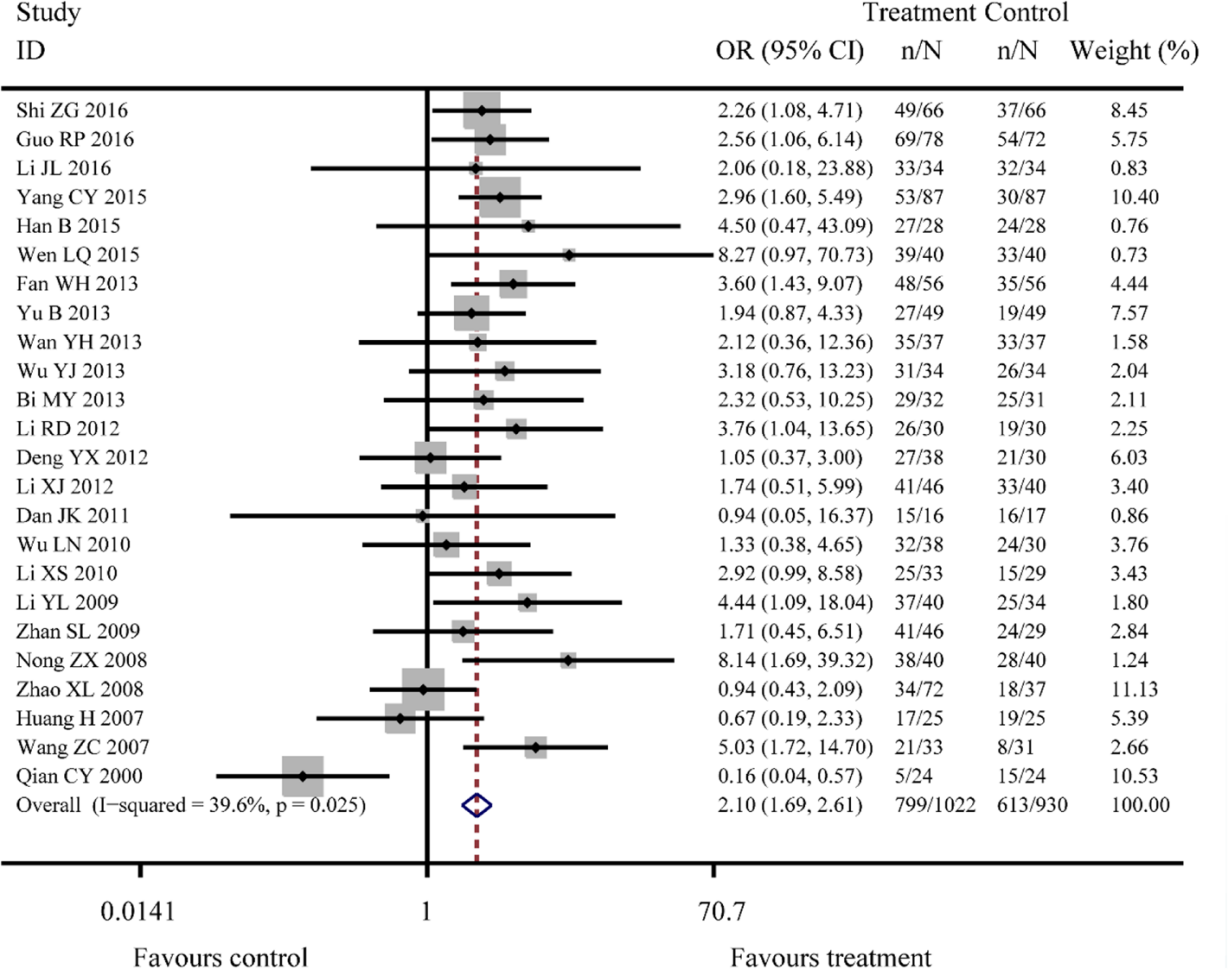
Meta-analysis of Hp eradication rates about two groups

**Fig. 4:**
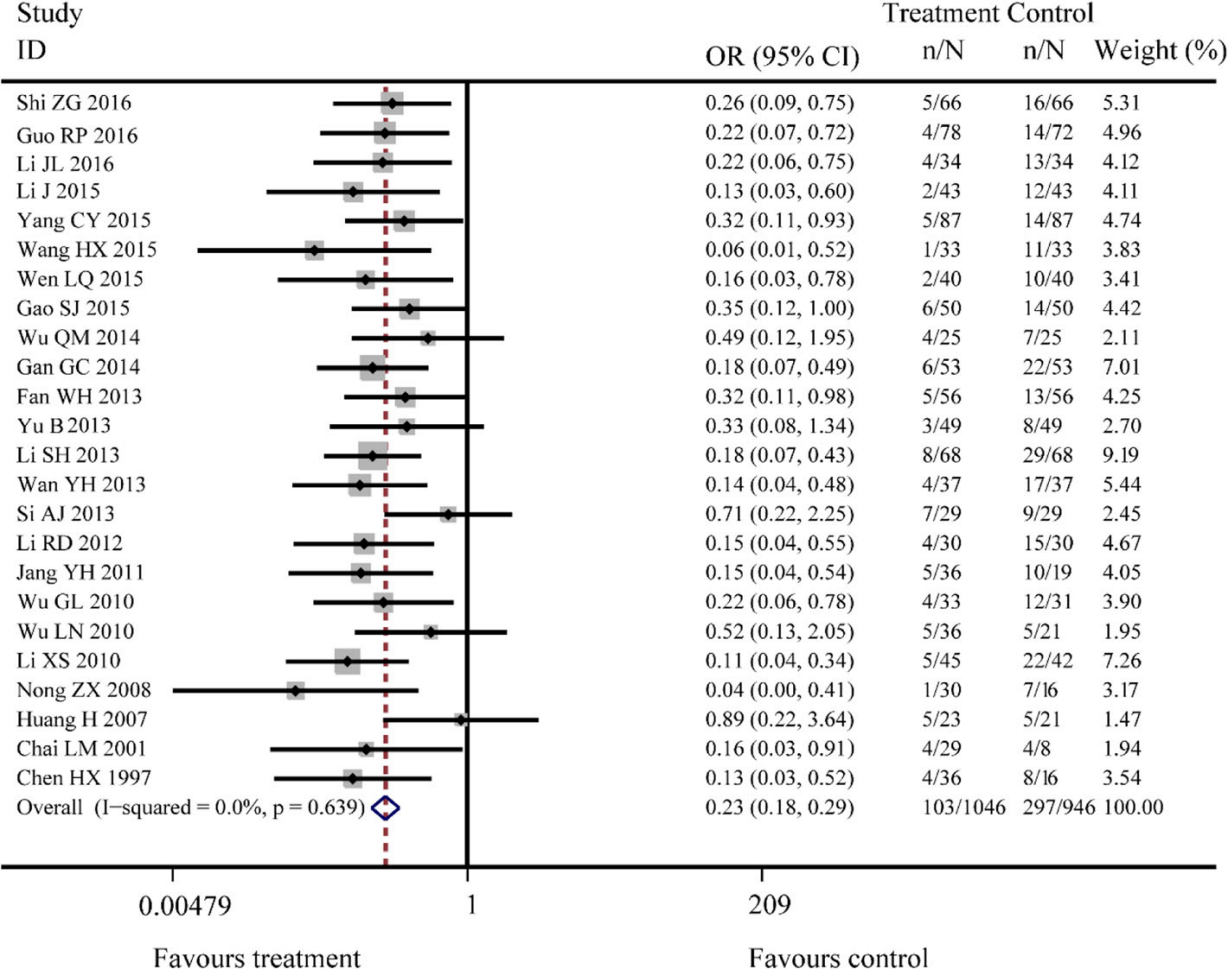
Meta-analysis of recurrence rates during two groups’ following-up

**Fig. 5:**
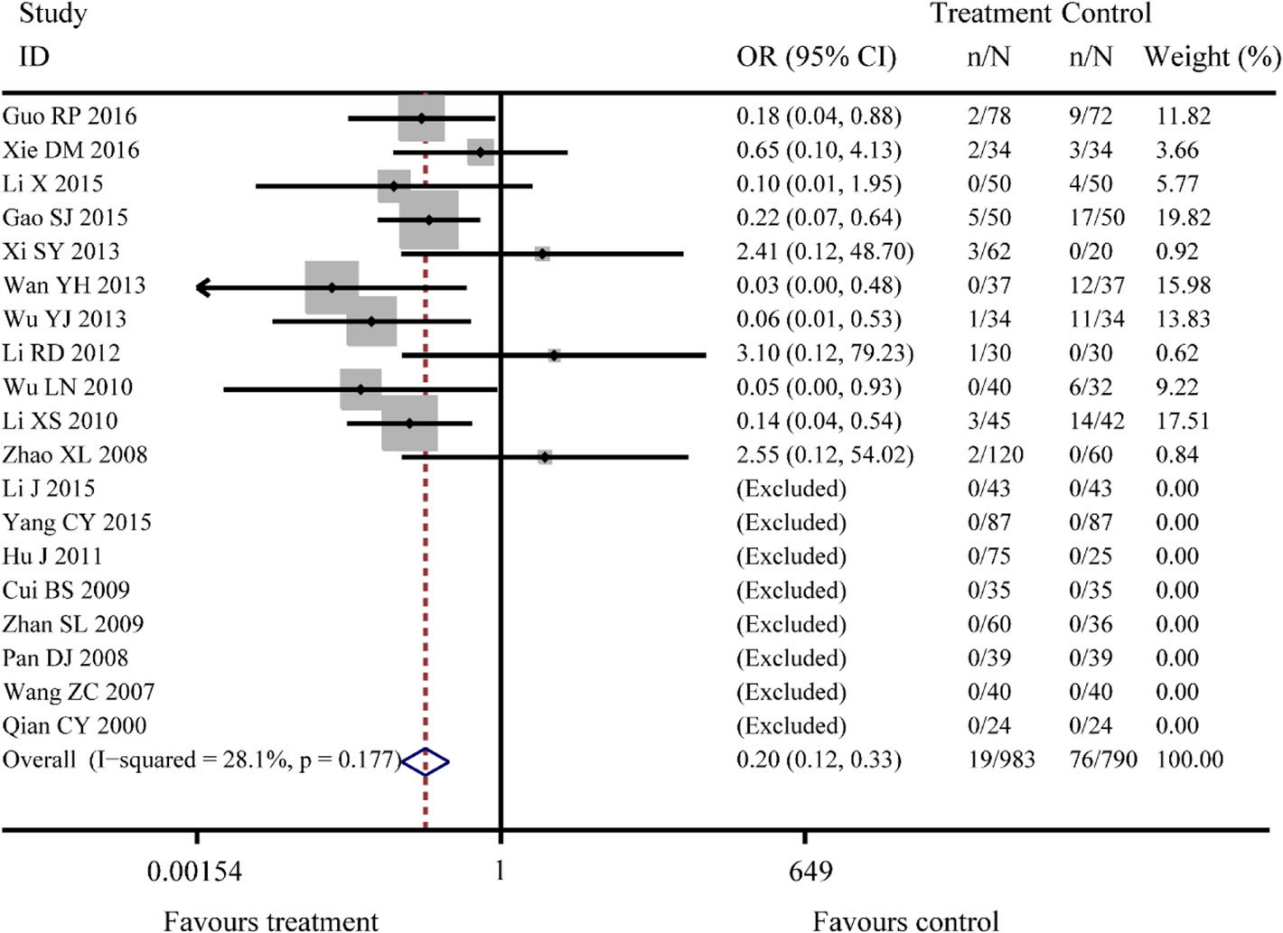
Meta-analysis of adverse reaction rates about two groups

Outcome indicators involved in all studies included the total effective rate, recurrence rate, Hp eradication rate, and adverse reaction rate; of these, 50 studies have reported the total effective rate. The results of the fixed effects model combined with effect sizes showed that the difference between Jianzhong decoction and conventional WM alone was significant in treating PU (OR =4.29, 95% CI: 3.51–5.23, *P* < 0.001, *I* ^*2*^ = 0.0%, *P* = 0.987; Fig. 2). Moreover, there were 24 studies reporting the Hp eradication rate and the results of fixed effects model combined with effect sizes showed a significant difference between Jianzhong decoction and conventional WM alone in treating PU (OR =2.10, 95% CI: 1.69–2.61, *P* < 0.001, *I* ^*2*^ = 39.6%, *P* = 0.025; Fig. 3). There were 24 studies reporting the recurrence rate and the results of fixed effects model combined with effect sizes showed a significant difference between Jianzhong decoction and conventional WM alone in treating PU (OR =0.23, 95% CI: 0.18–0.29, *P* < 0.001, *I* ^*2*^ = 0.0%, *P* = 0.639; Fig. 4). There were 19 studies reporting the adverse reactions and the results of fixed effects model combined with effect sizes showed a significant difference between Jianzhong decoction and conventional WM alone in treating PU (OR =0.20, 95% CI: 0.12–0.33, *P* < 0.001, *I* ^*2*^ = 28.1%, *P* = 0.177; Fig. 5).

### Sensitivity and publication bias analyses

In order to validate the robustness of the meta-analysis, we performed the sensitivity analysis on the comparison of the total effective rate, Hp eradication rate, recurrence rate, and adverse reactions. The exclusion of each study individually did not significantly alter the OR values of the total effective rate, Hp eradication rate, recurrence rate, and adverse reactions, indicating that the meta-analysis has a relatively high result stability and reliability. The detailed results are shown in Additional file 1: Figures S1–4.

**Fig. 6:**
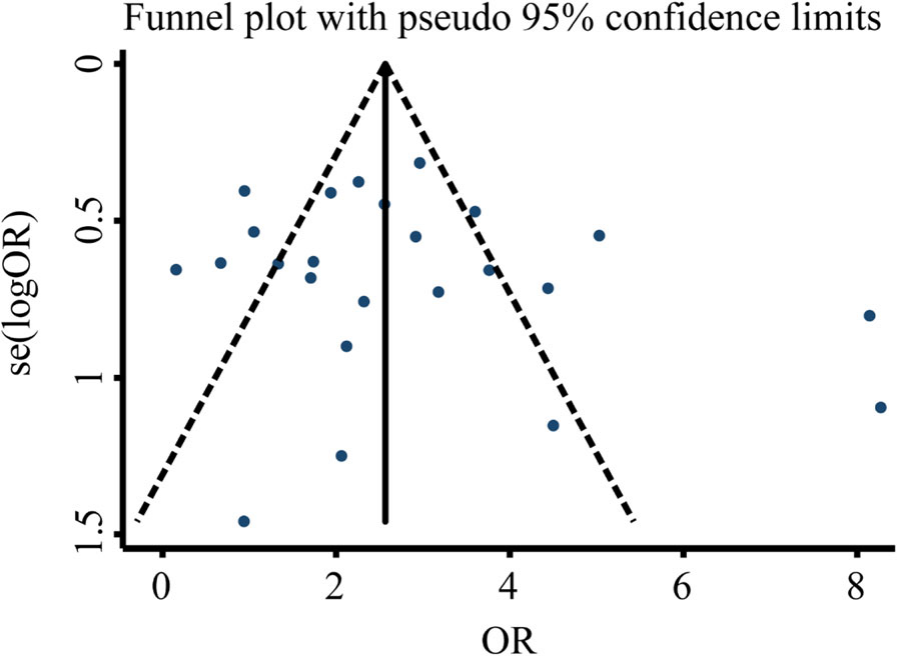
Inverted funnel plots of bias analysis on total effective rates

**Fig. 7:**
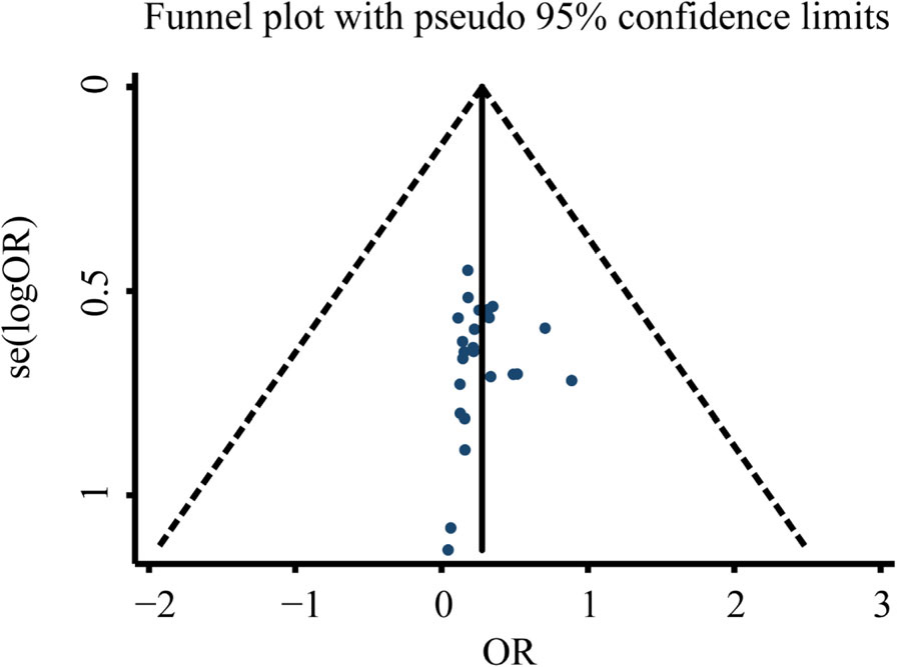
Inverted funnel plots of bias analysis on Hp eradication rates

**Fig. 8:**
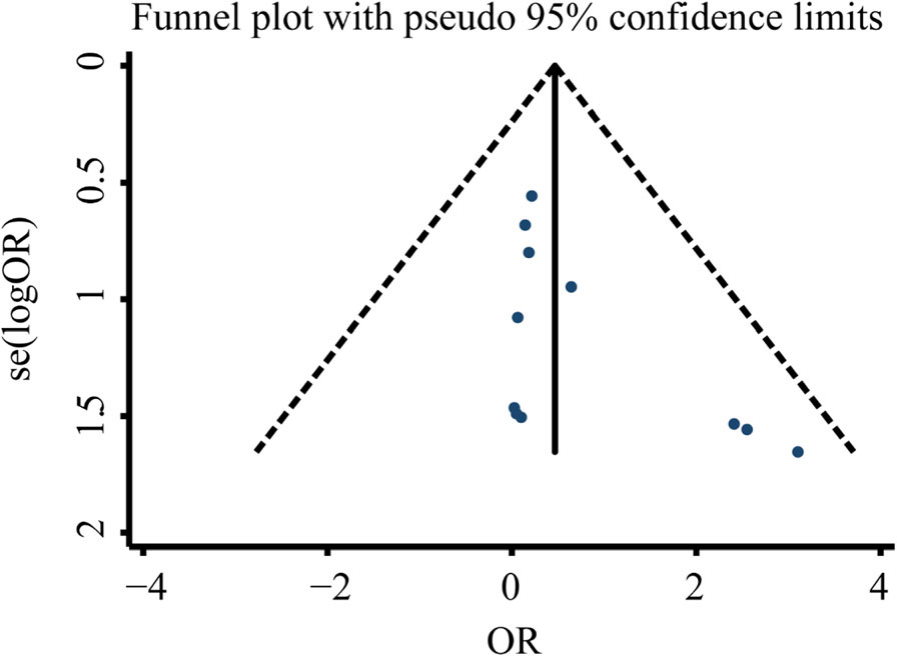
Inverted funnel plots of bias analysis on recurrence rates

**Fig. 9:**
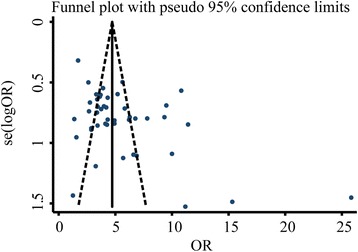
Inverted funnel plots of bias analysis on adverse reaction rates

We have analysed the publication bias of the aforementioned observation indicators and the results showed that the inverted funnel plots comparing the recurrence rate, Hp eradication rate, and adverse reactions were basically symmetrical as shown in Figs. 6, 7, and 8, respectively. The results of Egger’s test and Begg’s test also confirmed the absence of significant publication bias in those included studies: Hp eradication rate (Egger’s: *P* = 0.914; Begg’s: *P* = 0.785); recurrence rate (Egger’s: *P* = 0.213; Begg’s: *P* = 0.157); and adverse reaction rate (Egger’s: *P* = 0.518; Begg’s: *P* = 0.161). In contrast, the funnel plot comparing the total effective rate was asymmetrical, indicating the possible presence of publication bias in those included studies (Fig. 9). Moreover, the results of Egger’s and Begg’s tests also confirmed the presence of publication bias in those included studies reporting the total effective rate (Egger’s: *P* = 0.001; Begg’s: *P* = 0.202). The result robustness of the meta-analysis on the total effective rate was further analysed using the trim and filling method. After the addition of 15 false-negative unpublished studies, the results showed that the point estimation and 95% CI estimated value of the combined effect sizes did not change significantly before and after the clipping (before clipping: OR =4.06, 95% CI: 3.31–4.97, *n* = 47; after clipping: OR =3.29, 95% CI: 2.74–3.96, *n* = 62). This indicated that the meta-analysis on the total effective rate has a relatively better stability.

## Discussion

The PU is a result of synergistic damage caused by a gastric mucosal barrier dysfunction, enhanced gastric acid secretion, and Hp infection. Moreover, Hp infection is the main cause of PU with up to 80% and 90% of detection rate in gastric ulcers and duodenal ulcers, respectively. Therefore, an effective eradication of Hp has become the key to treat PU [9, 64, 65]. Presently, the triple and quadruple WM therapies are commonly used as the first-line clinical treatment program for managing PU. The WM-based therapies have the advantages of inducing rapid effects, rapid remission of clinical symptoms, high Hp eradication rates, and short ulcer healing times [66–68]. However, previous studies have reported several limitations of WM in treating PU, such as inducing severe side effects, increasing the patients’ susceptibility to adverse reactions, poor compliance, drug resistance, recurrence, and Hp infection [69, 70]. It has been also reported that patients have up to 50–70% of recurrence rates after 1 year of remission and the recurrence rates may increase up to 80–100% after 5 years [71]. Therefore, it is extremely urgent to search for a new ideal solution for the treatment of PU. The TCM Jianzhong decoction exerts various health benefits, such as “invigorate spleen and stomach”, “regulate qi and resolve the phlegm”, and “remove blood stasis and relieve pain”. Currently, Jianzhong decoction is widely applied in the treatment of PU with a satisfactory therapeutic efficacy. Indeed, previous studies have shown that Jianzhong decoction could effectively promote the healing of ulcers and improve the Hp eradication rate in the treatment of PU with only mild side effects and low recurrence rate [10]. However, the majority of the available clinical trials only involved a small sample size and a systematic evaluation of Jianzhong decoction therapeutic efficacy is still lacking. Therefore, the clinical efficacy of Jianzhong decoction in treating PU is still controversial.

In order to elucidate this, we analysed the overall therapeutic efficacy and safety of Jianzhong decoction against PU using meta-analysis of systematically retrieved RCTs. We have included a total of 58 studies that included 5192 patients. Our results showed that the therapeutic efficacy of Jianzhong decoction significantly outperforms that of the conventional WM in treating PU with respect to the total effective rate, Hp eradication rate, recurrence rate, and adverse reaction rate. Our findings indicated that Jianzhong decoction has a significant clinical efficacy in treating PU with low adverse reaction and recurrence rates.

Jianzhong decoction could “warm and tonify the deficiency” and “alleviate the related pains” in PU cases. It integrates various herbs that exhibit synergistic effects on the spleen deficiency-type PU by promoting the healing of ulcers and preventing their recurrence. In the TCM theory, middle-jiao (the middle part of a human body) is the key location for human digestion, and the pathogenesis of PU is the qi of middle-jiao hurt by cold-evil. The PU, especially with pain, can be treated by Jianzhong decoction. The name, Jianzhong, means construct the qi of middle-jiao. Clinically, TCM uses some combinations of sweet and warm-natured herbs as diverse kinds of Jianzhong decoction to treat different middle-jiao’s qi damage situations (such as short term or long term, slight damage or heavy damage). Here, the sweet and warm-natured herbs combinations are Sichuan pepper + Rhizoma zingiberis + Ginseng, Cassia twig + Chinese herbaceous peony + maltose, or Astragalus membranaceus + Cassia twig + Chinese herbaceous peony.

About the pharmacological aspects, these sweet and warm natured herbs can anti-inflammation, antihistamine, antibacterial, and protect gastric mucosa. Rhizoma zingiberis exerts distinct efficacy clinically. Research showed that it can induce macrophages to highly express inducible nitric oxide synthase, thereby synthesizing nitric oxide, which can mediate the activation of macrophages to influence the inflammation process [72]. Other research suggested that Ginseng polysaccharides promotes NK cell cytotoxicity in immunosuppressed mice by increasing the number of NK cells in the whole blood and upregulating the expression of perforin and granzyme. Thus, the research showed that Ginseng has a wide application prospects in the treatment of immunodeficiency diseases [73]. The major bioactive component of Rhizoma Zingiberis is Compound D, (E)-4-(3′, 4′-dimethoxyphenyl) but-3-en-1-ol, which has a strong smooth muscle relaxant, and has antihistamine and anti-inflammatory actions [74]. Ramulus Cinnamomi (Cassia twig), used to improve pharmacological effects and reduce toxicity [75]. Ginseng can inhibit H. pylori induced gastric inflammation by suppressing induction of inflammatory mediators, myeloperoxidase activity, and lipid peroxide level in H. pylori-infected gastric mucosa [76].

Jianzhong decoction combines various medications that not only could inhibit the secretion of gastric acid but also could enhance the expression of basic fibroblast growth factor (bFGF) in gastric mucosa of PU patients. Enhanced bFGF, thus, promotes the microvascular formation in the ulcers’ granulation tissue, thereby accelerating their repair and healing [6]. Studies have shown that Jianzhong decoction has a high healing rate and level of safety, as well as a very low recurrence rate in treated PU [11]. The result of an RCT showed that, when compared with the WM control group, Jianzhong integrated with Dansen decoction has a 94.4% of total effective rate, 88.5% of Hp eradication rate and 5.1% of 6-month recurrence rate in treating PU [9]. Another study has also reported a 94.1% of total effective rate following PU treatment with Jianzhong decoction, significantly exceeding that of the control group, and in the absence of any adverse reactions [10]. In line with these previous reports, we also demonstrated that Jianzhong decoction significantly outperforms the conventional WM in treating PU with respect to the total effective rate and Hp eradication rate. Moreover, Jianzhong decoction resulted in significantly lower rates of recurrence and adverse reactions compared with the conventional WM. The sensitivity analysis showed that the meta-analysis results of the total effective rate, Hp eradication rate, recurrence rate, and adverse reaction rate are robust and reliable. However, the total effective rate showed a significant publication bias. Hence, we carried out a further analysis using the trim and filling method by supplementing with similar studies and combining their effect sizes. We found that the point estimation and 95% CI estimated value of their combined effect sizes did not change significantly before and after the clipping, indicating that the meta-analysis results of the total effective rate are robust. The above-mentioned results suggest that Jianzhong decoction could effectively promote the healing of ulcers and alleviate the clinical symptoms in patients, in association with a high Hp eradication rate, low recurrence rate, and high level of safety in the treatment of PU.

Although Jianzhong decoction exhibited some benefit in PU treatment, there are a number of limitations to our study. Firstly, the majority of the studies had small samples. Secondly, the included studies were of low methodological quality. Finally, the majority of the included studies involved a short follow-up duration, leading to a generally low evidence for long-term using. Therefore, our observations still need to be validated by involving more high-quality, large-scale RCTs in future.

## Conclusion

In summary, Jianzhong decoction can improve the clinical symptoms of PU patients. So, Jianzhong decoction can be used as an ideal option for treating PU. However, quality evidence is needed to further assess its efficacy and safety.

## Additional file

Additional file 1: Sensitivity analysis on the comparison of the total effective rate, Hp eradication rate, recurrence rate, and adverse reactions. (DOCX 808 kb)

## Abbreviations

bFGF: basic fibroblast growth factor
CBMdisc: China biology medicine disc
CI: Confidence interval
CNKI: China national knowledge infrastructure
CSTJ: China science and technology
GI: Gastrointestinal
Hp: Helicobacter pylori
NSAIDs: non-steroidal anti-inflammatory drugs
OR: Odds ratio
PU: Peptic ulcer
RCTs: Randomised controlled trials
TCM: Traditional Chinese medicine
WM: Western medicine
