# Supplementary figures and images for: Efficacy and safety of Jianzhong decoction in treating peptic ulcers: a meta-analysis of 58 randomised controlled trials with 5192 patients
Source: BMC Complement Altern Med. 2017 Apr 14;17:215. doi: 10.1186/s12906-017-1723-2 (PMC5391578; doi:10.1186/s12906-017-1723-2)

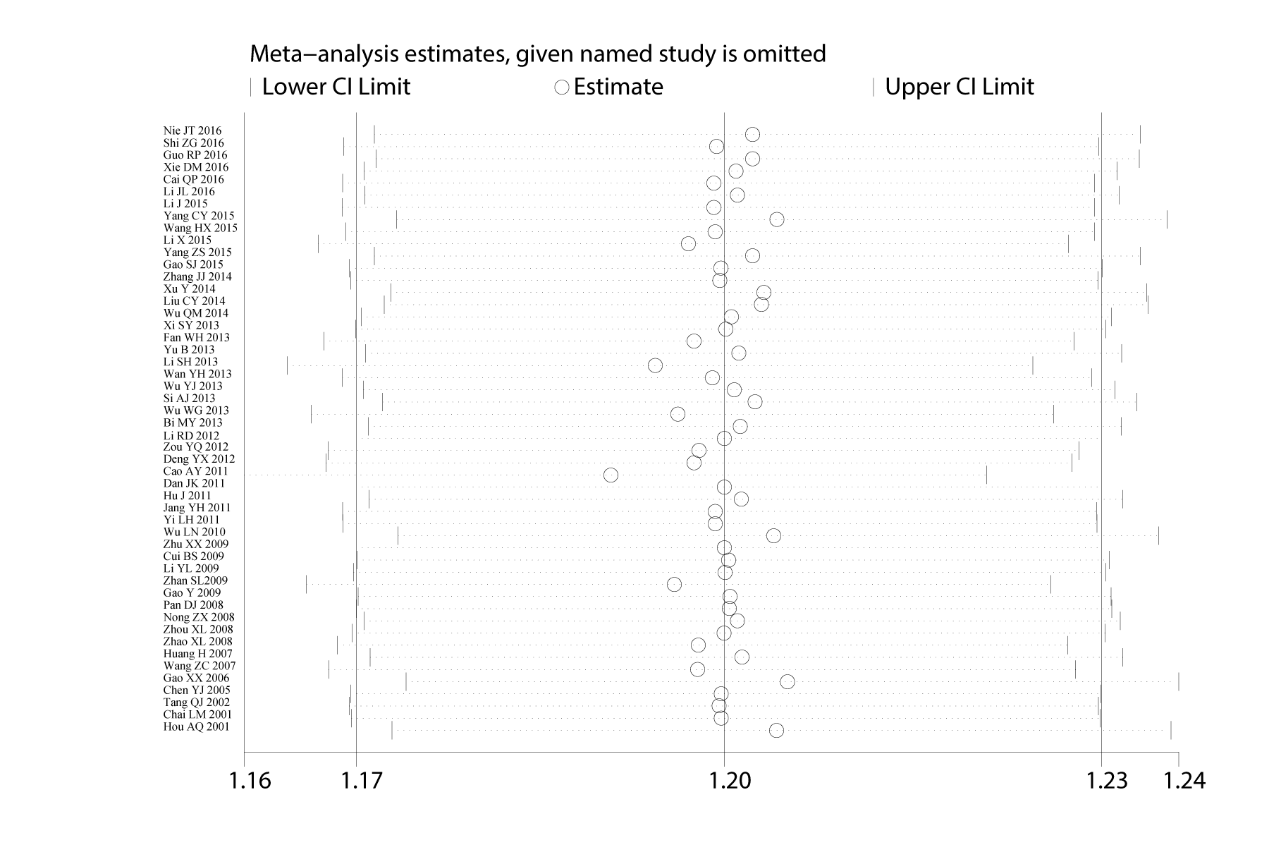


Figure S 1.


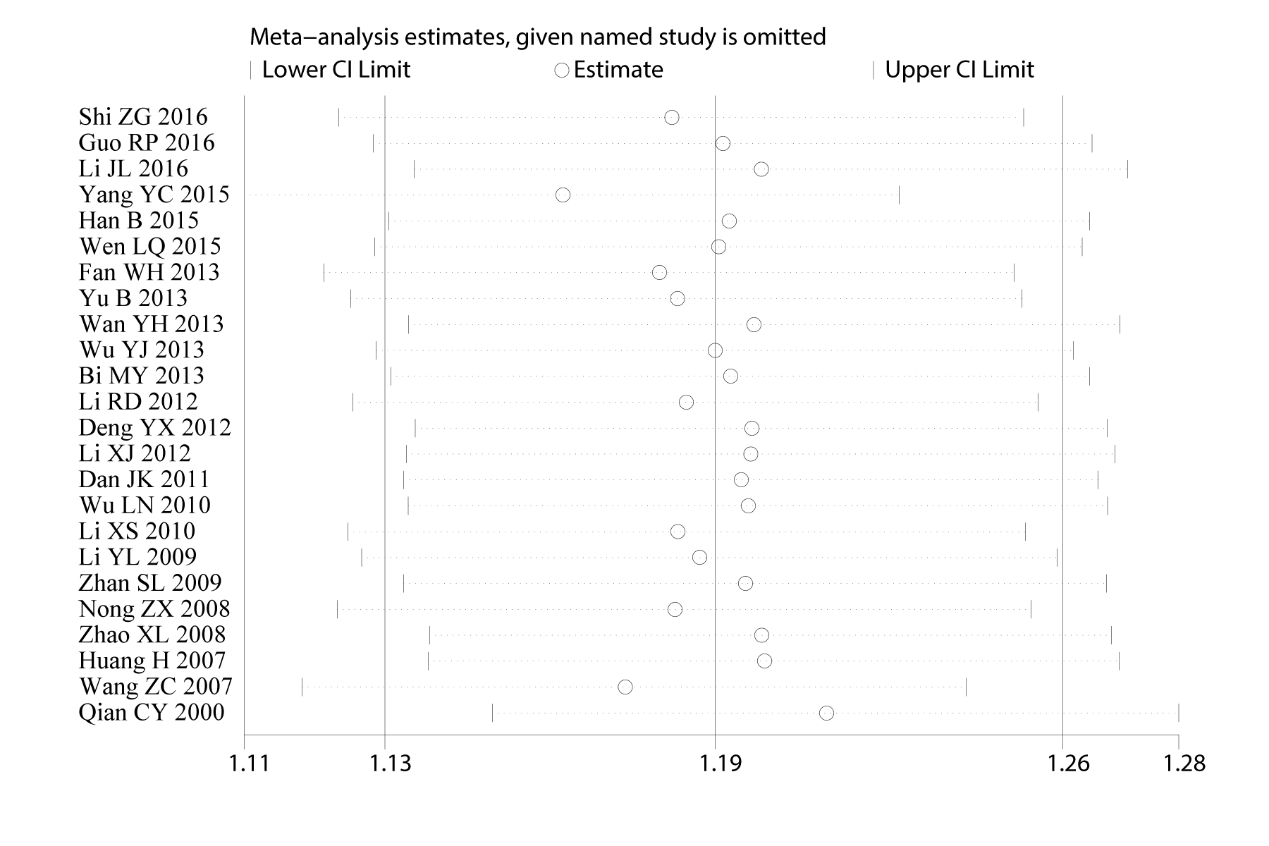


Figure S 2.


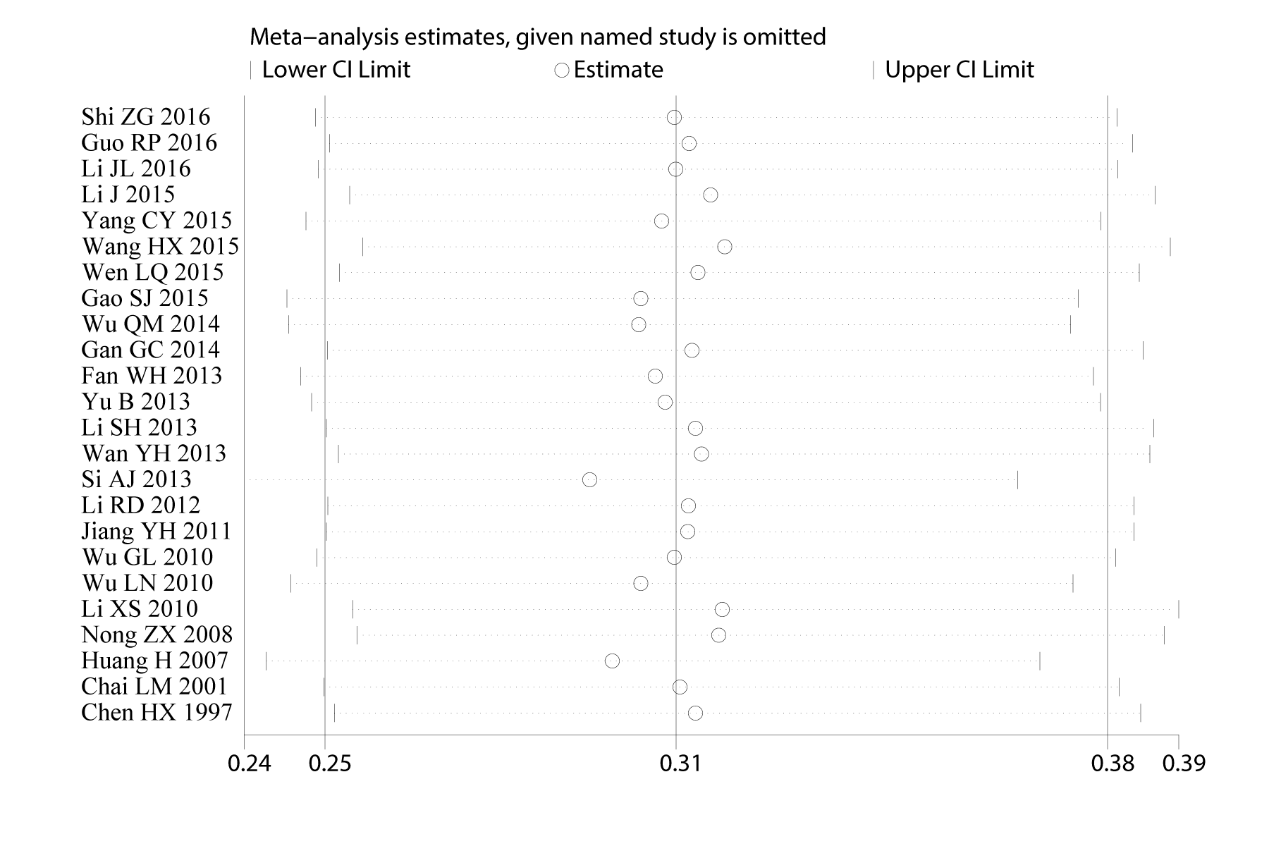


Figure S 3.


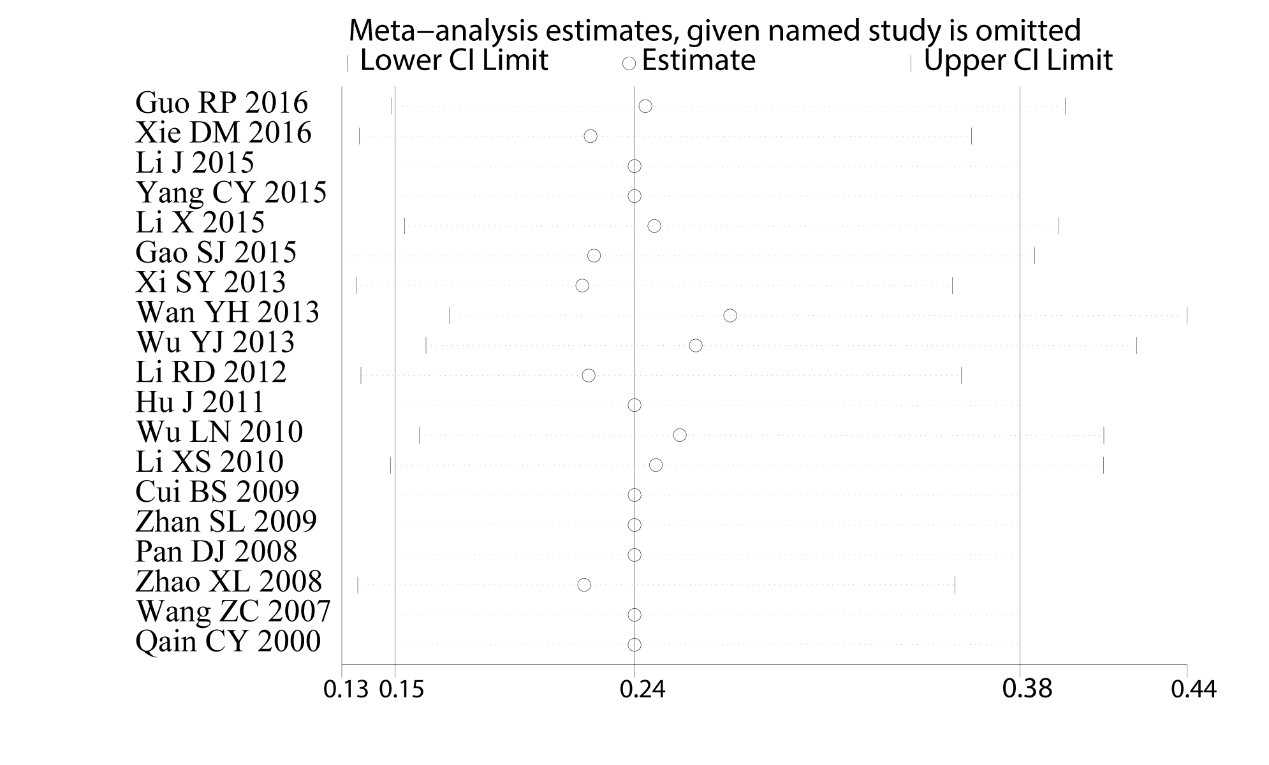


Figure S 4.

Supplement: Additional file 1: — Sensitivity analysis on the comparison of the total effective rate, Hp eradication rate, recurrence rate, and adverse reactions. (DOCX 808 kb) [file 12906_2017_1723_MOESM1_ESM.docx]
